# Correlation of Sedline-generated variables and clinical signs with anaesthetic depth in experimental pigs receiving propofol

**DOI:** 10.1371/journal.pone.0275484

**Published:** 2022-09-29

**Authors:** Alessandro Mirra, Claudia Spadavecchia, Olivier Levionnois

**Affiliations:** 1 Section of Anaesthesiology and Pain Therapy, Department of Clinical Veterinary Medicine, Vetsuisse Faculty, University of Bern, Bern, Switzerland; 2 Graduate School for Cellular and Biomedical Sciences, University of Bern, Bern, Switzerland; PLOS (Public Library of Science), UNITED KINGDOM

## Abstract

Most of currently available electroencephalographic (EEG)-based tools to assess depth of anaesthesia have not been studied or have been judged unreliable in pigs. Our primary aim was to investigate the dose-effect relationship between increasing propofol dose and variables generated by the EEG-based depth of anaesthesia monitor Sedline in pigs. A secondary aim was to compare the anaesthetic doses with clinical outcomes commonly used to assess depth of anaesthesia in this species. Sixteen juvenile pigs were included. Propofol infusion was administered at 10 mg kg^-1^ h^-1^, increased by 10 mg kg^-1^ h^-1^ every 15 minutes, and stopped when an EEG Suppression ratio >80% was reached. Patient state index, suppression ratio, left and right spectral edge frequency 95%, and outcomes from commonly used clinical methods to assess depth of anaesthesia in pigs were recorded. The best pharmacodynamic model was assessed for Patient state index, suppression ratio, left and right spectral edge frequency 95% in response to propofol administration. The decrease of Patient state index best fitted to an inhibitory double-sigmoid model (including a plateau phase). The increase of suppression ratio fitted a typical sigmoid E_max_ model. No relevant relationship could be identified between spectral edge frequency 95% values and propofol administration. A large variability in clinical outcomes was observed among pigs, such that they did not provide a reliable evaluation of propofol dose. The relationship between propofol dose and Patient state index/suppression ratio described in the present study can be used for prediction in future investigations. The evaluation of depth of anaesthesia based on common clinical outcomes was not reliable.

## Introduction

Despite the large use of the porcine model in translational medicine [1–3], appropriate objective methods to guarantee an adequate depth of anaesthesia (DoA) in pigs are still missing. This is not only a great ethical concern [4], but it seriously raises the question if the results obtained from translational studies are reliable.

Over the last 20 years, different variables to assess DoA have been investigated in pigs [5–16].

The SedLine monitor (Masimo Corp., CA, USA) has been recently released on the market. It is designed to record the electroencephalographic (EEG) signal in humans from left and right frontal and pre-frontal transcutaneous electrodes (RD SedLine EEG-sensor). The signal is processed through proprietary algorithms and the Patient State Index (PSI), the suppression ratio (SR) and the left and right 95% spectral edge frequencies (SEF 95%) are calculated. So far, its usability in pigs has been investigated [17] but the correlation between Sedline-generated variables and anaesthetic doses has not been reported.

Besides EEG-based tools, clinical variables (e.g. palpebral reflex, withdrawal reflex, jaw tone) are generally used in pigs to assess DoA [18–20]. However, the relationship between anaesthetic drugs and doses with the clinical outcomes has not been properly evaluated.

Most studies investigating DoA in pigs have been performed under inhalant anaesthesia. Therefore, only scarce information is available about propofol-based anaesthetic protocols and propofol effects on DoA variables in this species. As propofol is largely used in experimental pigs, the characterization of its dose-dependent effects is highly relevant to guarantee anaesthetic safety and animal welfare.

The primary aim of this study was to investigate the pharmacodynamic dose-effect relationship between propofol and PSI, SR and SEF 95% (left and right) response in pigs. A secondary aim was to compare clinical assessment of DoA with anaesthetic dose and the aforementioned Sedline-generated variables.

According to available data from the use of Sedline in humans, we hypothesized that the dose-effect relationship between propofol and Sedline-derived parameters (SR, SEF 95%, PSI) would follow a sigmoid Emax model [21–25].

We also hypothesized that clinical parameters would be largely heterogeneous among pigs, therefore not allowing a fixed relationship with Sedline-generated variables and increasing dose of anaesthetic.

## Materials and methods

A permission to perform the study was obtained from the Committee for Animal Experiments of the Canton of Bern, Switzerland (Protocol Number: 32015). Each pig underwent a single experimental trial, at the end of which it was euthanized (pentobarbital; 150 mg kg^-1^ intravenous (IV)). All efforts were made in order to minimize suffering. Sample size calculation was performed for another investigation running on the same animals and evaluating two groups during the recovery phase without interference with the present study.

### Animals

Sixteen pigs (phenotype Edelschwein) of both sexes (10 females and 6 males) with a body weight of 28.9± 4.7 kg (mean±standard deviation (SD)) and of 10±0.6 weeks of age were included in the study.

Pigs were collected from the farm of origin between two and ten days before the experiment in groups of at least two animals and brought to the animal facility of the University of Bern. The animals were housed in single boxes. Visual and auditory contact was allowed during the whole stay, and at least three pigs were always present in the facility. Clinical examination was performed once a day by a veterinarian. The animals were fed three times per day and had ad libitum access to water.

### Instrumentation

On the experimental days, the animals were briefly acclimatized to the experimental room (between 30 and 60 minutes) and then placed into a sling for instrumentation. A local anaesthetic cream (eutectic mixture of local anesthetics (EMLA) 5%, Anesderm, Pierre Fabre, Switzerland) was applied over the two ears and on the tail for at least 45 minutes before placing venous (auricular) and arterial (auricular or coccygeal) catheters. The dorsal part of the skull was prepared and the pediatric RD SedLine EEG-sensor placed as previously described [17].

### Treatment

A propofol (Propofol 1% MCT, Fresenius Kabi AG, Switzerland) infusion was started at 10 mg kg^-1^ h^-1^ and then increased by 10 mg kg^-1^ h^-1^ every 15 minutes. Oxygen supplementation was always provided via face mask. Endotracheal intubation (ETI) was performed as deemed appropriate by the anaesthetist (AM). After ETI, volume-controlled mechanical ventilation was provided with a tidal volume of 15 ml kg^-1^ while the respiratory rate was adjusted targeting an end-tidal carbon dioxide partial pressure (EtCO_2_) between 4.6 and 5.9 kPa (35 and 45 mmHg).

The propofol infusion was increased until the EEG suppression ratio (SR, as read on the Sedline monitor) was maintained above 80% for continuative10 minutes. At that point, the infusion was stopped and the pig allowed to recover.

At least 90 minutes later, the pig was euthanised with pentobarbital IV (150 mg kg^-1^).

### Data collection

Data generated by the Sedline device (PSI, SR, SEF 95%-left ad -right) were recorded and saved automatically every 2 seconds until euthanasia. Eye position was graded every 5 minutes as “central”, “rotated” or “nystagmus”. Palpebral reflex and jaw tone were assessed manually once at baseline (before propofol start), and then every 5 minutes. Both were scored each time as 2 (same as baseline), 1 (reduced) or 0 (absent). At the same time points, mechanical and thermal nociceptive stimulations were applied sequentially. A spring clamp (Little Samson; Salter Abbey, West Midlands, UK) was attached to one arm of a metallic clamping tong and was used to perform the mechanical stimulation. The stimulus was applied at the level of the third or fourth digit of the left hind limb, alternating them to avoid hypersensitization. A constant force of 60 Newtons was applied until a withdrawal response appeared, or for a maximum of 60 seconds. The response was scored similarly as above (score from 2 (same as baseline) to 0 (absent)). Additionally, the sum of the scores for palpebral reflex, jaw tone and response to mechanical stimulation was recorded as the clinical depth of anaesthesia score (CDS, score from 6 to 0). The thermal stimulation was performed using an Nd-YAG (neodymium doped yttrium–aluminum–garnet) laser (Stimul 1340, Deka, Italy), wavelength 1.34 μm. Pulses of 5 joules (J), 20 milliseconds (ms) duration and 10 mm spot diameter, were applied to the previously shaved lateral aspect of the left front limb, at a 10 cm distance from the skin. The response to laser stimulation was scored as 2 (present at 5J), 1 (present only if increased at 10 or 15J) or 0 (absent at 15 J). At least 30 seconds elapsed between stimuli to avoid temporal summation, and the stimulation site was changed slightly to avoid hypersensitization.

### Data analysis

For data analysis, the individual durations of propofol administration were divided in 30 equal time intervals (TI) for each pig (TI_1_ to TI_30_). With this transformation, it was assumed that DoA was increasing continuously from TI_1_ to TI_30_ and that all the pigs had approximately similar DoA at the same TI. For each TI, individual means for PSI, SR and SEF 95%-left and -right, and individual CDSs were calculated. Baseline values (TI_0_) for each parameter were also calculated, considering a recording time of an equal length of a single TI, for each pig, taken before propofol start.

Differences between SEF 95% -left and -right were tested for significance with a two-way ANOVA test for repeated measures. Correlations between pairs of variables for propofol dose, PSI, SEF 95% -left, SEF 95%-right and CDS were tested with the Spearman’s rank correlation coefficient (SigmaStat 10, Systat Software Inc., United States).

### Pharmacodynamic modeling

A pharmacodynamic direct-effect model was established for PSI, SR, SEF 95%-left and -right in response to propofol administration using the software Phoenix 64 v.8.3. NLME (Pharsight Inc., United States).

First, the dose-effect relationship was visually inspected to select possible models to be used among linear, polynomial, exponential (E_max_), sigmoidal or double-sigmoidal [26, 27], and to determine whether data-transformation was required. Then candidate models were compared by visual analysis of goodness-of-fit (GOF) plots, -2LL significance (p < 0.05) and Akaike information criterion (AIC)/ Bayesian information criterion (BIC) for nested models. The best final model was determined with a population pharmacokinetic (popPK) approach using quasi-random parametric expectation maximization (QRPEM) with combined additive and multiplicative error model of residual unexplained variability (RUV). Random effect was tested on PD parameters using exponential model for intra-individual variability (IIV). Standard validation methods in popPK were used to assess model adequacy (visual predictive check and bootstrapping on 1000 replicates). Proportional prediction correction was applied for the visual predictive check (VPC).

## Results

Of the initial 16 pigs included in this study, seven were excluded from analysis. Three of them developed sudden respiratory obstruction before ETI. While obstruction was promptly resolved, alteration of the Sedline-generated variables was observed; therefore, these animals were excluded to avoid erroneous pharmacodynamic modeling. All the other pigs completed the entire experiment without complications. In four of them, Sedline data were not properly saved by the device, such that the animals were excluded. Thus, data are presented for nine pigs.

Marked spontaneous breathing was generally maintained after endotracheal intubation impairing the efficiency of mechanical ventilation. Median (interquartile ranges) EtCO_2_ between endotracheal intubation and the end of propofol administration was 8.9 (7.8–10.1) kPa [67 (59–76) mmHg].

**Fig 1:**
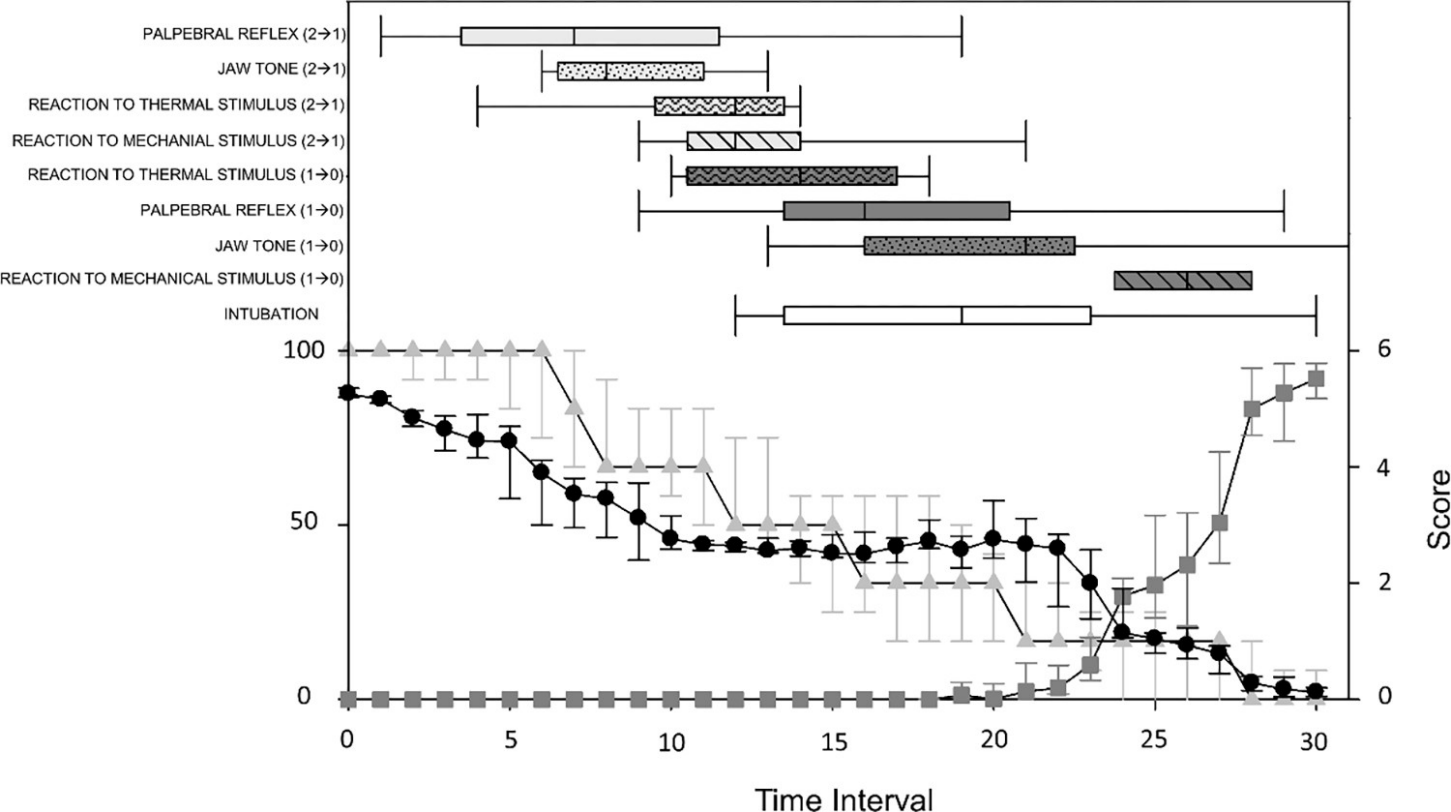
Sedline-generated and clinical variables over time. Median (interquartile range) for patient state index (black dots), suppression ratio (dark grey squares), and clinical depth of anaesthesia score (light grey triangles) during increasing propofol infusion in nine pigs. The individual durations of propofol administration were divided in 30 equal time intervals (TI) for each pig (TI_1_ to TI_30_). T_0_ = baseline values. Box plots (median and 25-75th percentiles) represent the time intervals at which the different clinical outcome scores changed (in brackets).

Median and interquartile ranges for propofol doses and TIs, corresponding to the moment in which the score of single clinical parameters changes (i.e., from 2 to 1 or from 1 to 0), are presented in Table 1. Values of PSI, SR, CDS and clinical parameters are represented in Fig 1.

**Table 1:** Median (interquartile ranges) of propofol doses (in mg kg^-1^ h^-1^) and time intervals at which the different clinical outcome scores changed (in square brackets) and intubation was performed.

EVENTS	Propofol dose	Time interval
	(mg kg-^1^ h^-1^)	
Palpebral reflex [2→1]	20 (15–30)	7 (3.5–11.5)
Palpebral reflex [1→0]	40 (50–55)	16 (13.5–20.5)
Jaw tone [2→1]	20 (20–30)	8 (6.5–11)
Jaw tone [1→0]	50 (45–60)	21 (16–22.5)
Clamp reaction [2→1]	30 (25–40)	12 (10.5–14)
Clamp reaction [1→0]	70 (60–80)	26 (24.5–29.5)
Laser reaction [2→1]	25 (12.5–30)	12 (9.5–13.5)
Laser reaction [1→0]	40 (30–40)	14 (10.5–17)
Intubation	50 (40–60)	19 (13.5–23)

The eye position was observed to be very variable within individuals and among pigs such that a trend could not be determined. The data on eye position were discarded. Palpebral reflex reduction/disappearance was extremely heterogeneous (in one pig started to be reduced at TI_1_, while in another it disappeared only at TI_29_). A similar pattern was noticed for jaw tone, that was still present (albeit reduced) in six pigs at the time of tracheal intubation. Five pigs (55%) stopped to react to thermal stimulation abruptly (the score decreased directly from 2 to 0); at TI_18_, all pigs had ceased to move in reaction to thermal stimulation. All pigs showed a progressive fading of the reaction to mechanical stimulation; two pigs never completely lost the reaction (score of 1) even at SR>80%.

**Fig 2:**
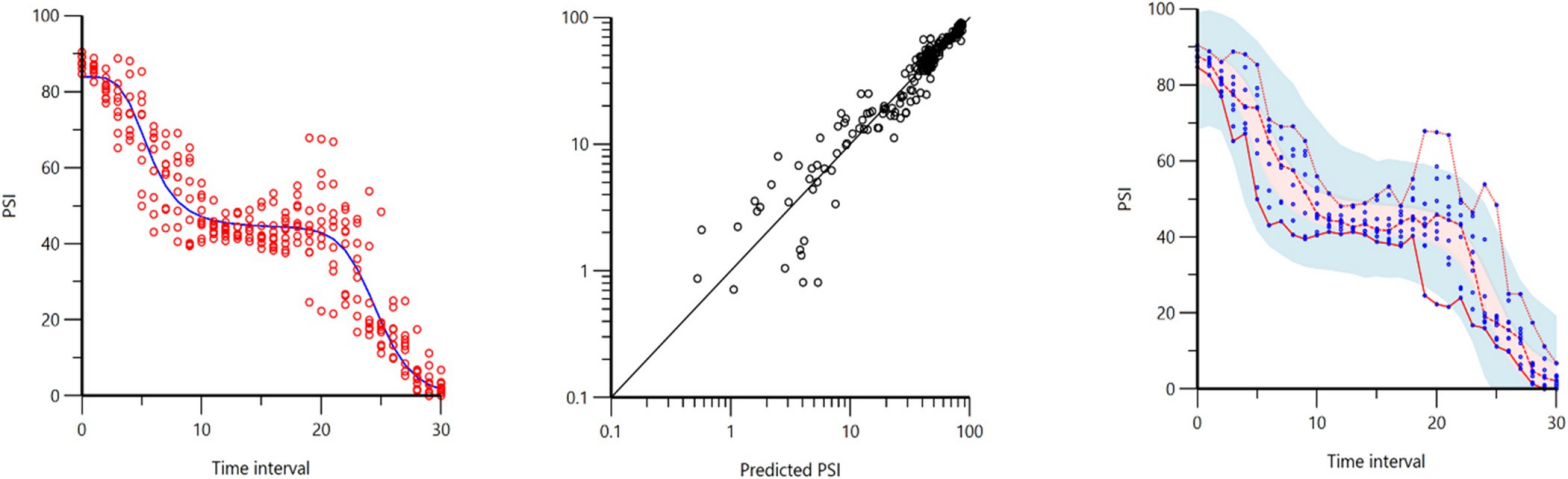
Pharmacodynamic modeling for patient state index (PSI). A. Observed data (circles) and prediction from the final population model (line) for PSI values during increasing propofol infusion rate. The time axis shows the normalized time intervals. B. Observed PSI data against individual-predicted values. C. Prediction-corrected visual predictive check (for 1000 simulations) of the final model for PSI; Blue dots = observations; red lines = 5^th^, 50^th^, and 95^th^ percentiles of the observed values; red/blue shaded area = 95% CI of the 5^th^, 50^th^ and 95^th^ percentiles of the final prediction model.

The PSI decreased over the course of the experiment (Fig 1). Initial PSI (TI_0_) was 87 [86–89] and reached the value of 1 [0–4] with a SR > 80% (TI_30_). Only three pigs (25%) maintained a PSI > 5. A large variability among individuals was observed between TI_18_ and TI_26_. The PSI-decrease fitted at best to an inhibitory double-sigmoidal model compared to linear or inhibitory sigmoidal (Fig 2). The final model (Eq 1) included fixed effect and random effect on E_max 1_, median effective dose (ED_50_) _1_, γ_1_, E_max 2_, ED_50 2_, and γ_2_ (Table 2).


$$E=(E_{{max}_{1}}*\frac{D^{\gamma_{1}}}{{{ED}_{50_{1}}}^{\gamma_{1}}+D^{\gamma_{1}}})+(E_{{max}_{2}}*\frac{D^{\gamma_{2}}}{{{ED}_{50_{2}}}^{\gamma_{2}}+D^{\gamma_{2}}})$$ (Eq 1)

**Table 2:** Pharmacodynamic parameter estimates and confidence interval for the final model and Bootstrap (1000 simulations) for patient state index (PSI) and suppression ratio (SR).

	Final model		Bootstrap	
	Estimate	Confidence interval 2.5–97.5%	Estimate	Confidence interval 2.5–97.5%
**Patient State Index (PSI)**				
E_max 1_	39.8	34.2–45.3	39.7	36.6–42.1
Median effective dose _1_	44.2	41.7–46.7	44.2	41.9–46.5
γ_1_	4.4	2.4–6.5	4.5	3.6–5.4
E_max 2_	5.6	3.7–7.6	5.7	4.7–6.8
Median effective dose_2_	16.3	2.0–30.6	16.7	12.3–22.3
γ_2_	24.6	22.4–26.9	24.6	24.0–25.2
**Suppression Ratio (SR)**				
Median effective dose	26.3	25.4–27.2	26.3	25.7–26.9
γ	18.2	11.5–24.9	18.4	13.3–25.1

**Fig 3:**
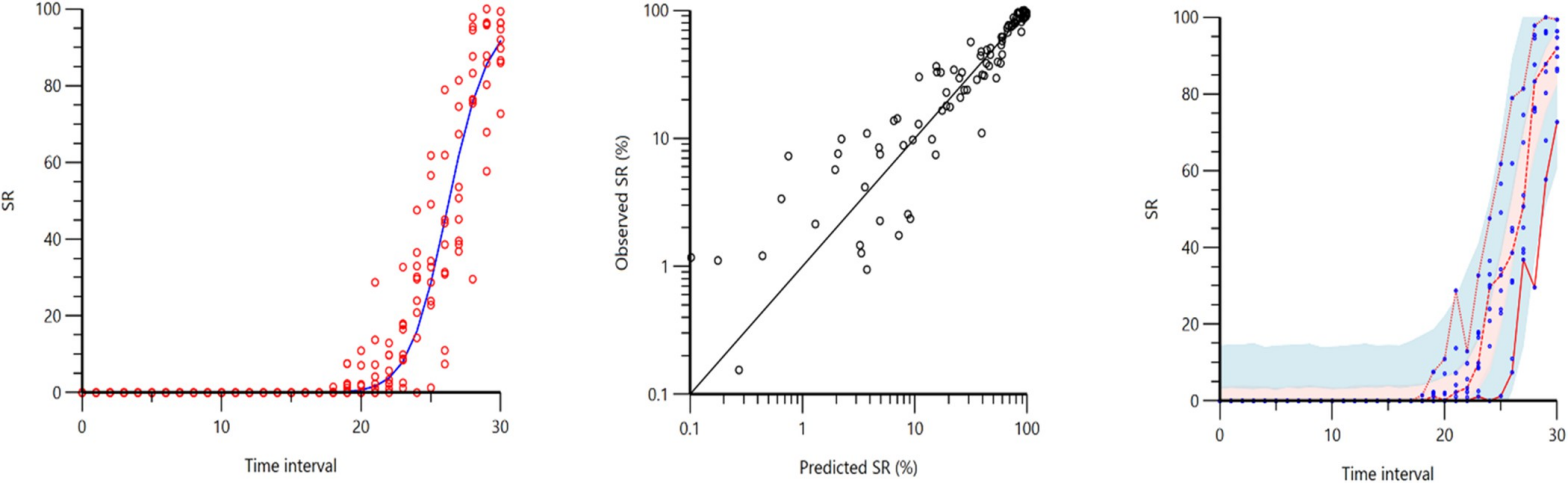
Pharmacodynamic modeling of suppression ratio (SR). A. Observed data (circles) and prediction from the final population model (line) for SR during increasing propofol infusion rate. The time axis shows the normalized time intervals. B. Observed SR data against individual-predicted values. C. Prediction-corrected visual predictive check (for 1000 simulations) of the final model for SR; Blue dots = observations; red lines = 5^th^, 50^th^, and 95^th^ percentiles of the observed values; red/blue shaded area = 95% CI of the 5^th^, 50^th^ and 95^th^ percentiles of the final prediction model.

The SR started to increase at TI_18_, followed by a steep increase (Fig 3). The SR-increase fitted a typical sigmoid E_max_ model (Fig 3). The final model included a constant E_max_ (100) and a fixed and random effect on ED_50_ and γ (Table 2).

**Fig 4:**
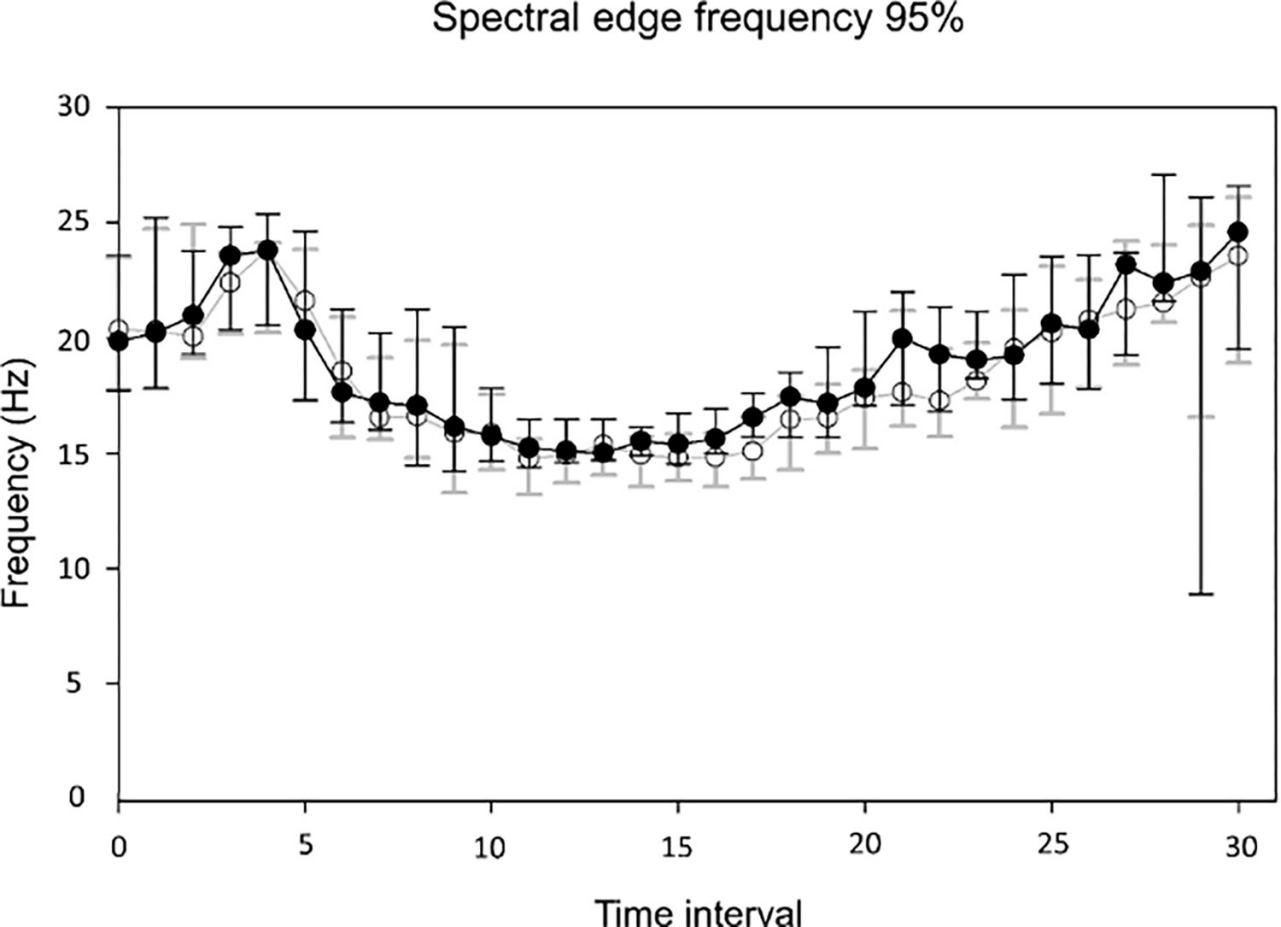
Spectral edge frequency 95% (median and interquartile ranges) -left (white dots) and -right (black dots) at each time interval (TI).

The values for left and right SEF 95% were not significantly different (p = 1.0, repeated measure two-way ANOVA). The relationship between SEF 95% values and propofol dose did not appear to follow an obvious trend at visual inspection (Fig 4). No modeling was performed.

Spearman correlation coefficients between pairs of variables for propofol dose, PSI, SEF 95%-left, SEF 95%-right and CDS are presented in Table 3.

**Table 3:** Spearman correlation coefficients (r_s_) measured for the relationship between propofol dose [corresponding to time intervals (TI)], patient state index (PSI), spectral edge frequency 95% (SEF 95%)-left and -right and clinical depth of anaesthesia score (CDS) in pigs under propofol anaesthesia.

r_s_	PSI	SEF 95%-left	SEF 95%-right	CDS
**Time interval (TI)**	-0.93	0.11	0.16	-0.98
**PSI**		0	0	0.9
**SEF 95%-left**			0.95	-0.09
**SEF 95%-right**				-0.14

## Discussion

In the present study we found that the relationship between PSI values and increasing dose of propofol is best represented by a double-sigmoid model, while that the relationship between SR and propofol dose is best represented by a sigmoidal model. No clear trend has been identified for SEF 95, that seems to have a poor performance in assessing DoA in pigs if used alone. As expected, the sequence with which the clinical parameters became depressed by increasing doses of propofol seemed to follow a common pattern, but there was a marked individual variation for each single parameter supporting that they cannot be used alone to evaluate DoA in pigs.

In previous publications [24, 28, 29], an inhibitory sigmoidal model was used to describe the relationship between PSI and propofol dose in humans. However, other investigations performed under inhalant anaesthesia found that the dose-effect relationship of EEG-derived variables obtained from BIS and Narcotrend devices was better described with the combination of two fractional sigmoid E_max_ models [26, 27, 30, 31]. The same observation was made for PSI in pigs undergoing propofol anaesthesia in the present study. Compared to linear, a sigmoid relationship displays higher sensitivity over the range covered by the steep parts of the curve. Simultaneously, the plateau phase of the curve may help the anaesthetist in maintaining DoA levels around a targeted range. In the present investigation, the plateau was observed at PSI value of 44 (E_max 2_ = 44.2), which falls within the range generally targeted during surgical anaesthesia in humans (25–50). Former studies reported a plateau at around 40 for BIS and 50 for Narcotrend [26, 27, 30, 31]. The value at which these indexes will tend to display a plateau over the range of anaesthetic doses tested depends on the computation algorithm, which is proprietary and often not published. Therefore, it is not possible to hypothesize if this is done on purpose and if it can be extrapolated to pigs.

Former publications in humans reported the plateau to appear at anaesthetic doses at which EEG suppression ratio starts to increase [26, 27, 30, 31]. In the present study, the plateau phase was displayed earlier (approximately at TI_15_) than appearance of SR (approximately TI_18_).

These results highlight how a mere translation of end-points set for humans to other species can lead to misleading results and wrong decisions. In order to ensure a correct and stable DoA, species-specific investigations should be performed and species-specific end-points set.

The present study supports previous reports describing SR dose-response relationship with a sigmoidal model [25]. Targeting a certain level of burst suppression has been described in the literature as effective way to guarantee deep levels of anaesthesia in pigs [5, 32]. However, it remains unclear if a specific ratio of EEG suppression can be used to differentiate anaesthetic planes more precisely than its sole presence/absence. In the present investigation, SR increased very rapidly from 10% to >80% over a narrow range of propofol doses. Most of the clinical signs commonly associated with inadequate DoA (e.g., presence of palpebral reflex, jaw tone, reaction to clamp) were still present when EEG suppression started to appear. However, this may be different when other drugs are administered. In humans, recent studies suggest avoiding the phase of EEG suppression during surgery to reduce the occurrence of post-operative delirium [33, 34]. No information regarding the possible influence of intraoperative SR on recovery outcomes is available in veterinary species, and further studies are needed to address this issue.

No obvious relationship was observed between SEF 95% and DoA. The SEF 95% has been widely described in a large variety of species to decrease with deepening of anaesthesia [35] until a paradoxical increase may happen when EEG suppression appears [36]. In the present study, SEF-95% initially increased, then decreased until reaching a plateau level, and finally started to strongly increase again, approximately when SR occurred. Such complex patterns may be the result of technical, methodological or physiological processes and would require further investigation.

Clinical DoA assessment still remains a cornerstone in veterinary anaesthesia. However, few investigations have been conducted to validate species-specific DoA scales, as well as the influence of different drugs on these clinical parameters. In the present investigation, the sequence of their depression tended to follow a reproducible pattern where reaction to thermal stimulation disappeared first, followed by palpebral reflex, jaw tone and finally reaction to mechanical stimulation. However, there was a large variability between subjects for the predicted DoA at which these events occurred. The variability was lower when evaluation of palpebral reflex, jaw tone and response to mechanical stimulation were combined in a score value (CDS). The CDS showed a potential for prediction of DoA, as well as PSI and SR values. Of notice, palpebral reflex, jaw tone and reaction to clamp stimulation were often present at PSI between 25 and 50.

The absence of reaction to a clamp has been previously associated with a deep anaesthetic plane in pigs [16, 18]. The reason why such a response was still present at high doses of propofol (SR>80%) in our study is not clear. Differences in methodology to apply the mechanical stimulation as well as the use of concomitant drugs with analgesic properties limit the comparison between published studies. In contrast to the response to mechanical stimulation, the motor reaction to thermal stimulation disappeared early in all the pigs. However, the different stimulation paradigm used (continuous pressure up to 60 seconds for the mechanical versus 20 ms single pulse for the thermal) must be taken in consideration, since it influences the response [14].

The PSI in the awake phase had a median value of 87. This is in line with what has been described in humans when it was assessed in awake patients, with and without closed eyes [37]. The calm behaviour of the animals once placed in the slings (sometimes also associated with a sleeping behaviour) could have prevented higher values being reached. Moreover, the brain areas covered by the recording electrodes may be different between human and pigs [17], influencing the calculated variables.

Incomplete saving of the SedLine-generated data has been already reported [17]; thus, it may be suggested to manually record the displayed values as well.

This paper has some limitations. First, the rate of propofol administration did not reflect how anaesthesia is provided in clinical practice. Different results may have been obtained using faster propofol administration and the use of concomitant drugs. Second, various biological signals have been reported to interfere with EEG recording activity (e.g. arterial pulse, electromyographic signals) [38, 39] and it may have also happened here. Third, the development of hypercapnia may have interacted with DoA and EEG activity, which could have modified the propofol dose-effect relationship [40]. Fourth, no sample size calculation was performed for the present study, being part of another investigation having a different primary aim.

## Conclusion

The relationship between propofol dose and PSI/SR described in the present study can be used for prediction in future investigations. No clear trend was identified between SEF 95% and increasing propofol doses. Based on the present findings, the use of single clinical outcomes to assess DoA in pigs undergoing propofol anaesthesia can be misleading, and should be avoided.
