# Correction: Correlation of Sedline-generated variables and clinical signs with anaesthetic depth in experimental pigs receiving propofol

**DOI:** 10.1371/journal.pone.0308594

**Published:** 2024-08-06

**Authors:** Alessandro Mirra, Claudia Spadavecchia, Olivier Levionnois

In Table 2, the data in the columns Final model and Bootstrap of rows "Median effective dose, E_max2_, Median effective dose_2_ and γ_2_ under Patients State Index (PSI) category are incorrect. Please see the correct Table 2 here.

**Table 2:** Pharmacodynamic parameter estimates and confidence interval for the final model and Bootstrap (1000 simulations) for patient state index (PSI) and suppression ratio (SR).

	Final model		Bootstrap	
	Estimate	Confidence interval 2.5–97.5%	Estimate	Confidence interval 2.5–97.5%
**Patient State Index (PSI)**				
E_max 1_	39.8	34.2–45.3	39.7	36.6–42.1
Median effective dose _1_	5.6	3.7–7.6	5.7	4.7–6.8
γ_1_	4.4	2.4–6.5	4.5	3.6–5.4
E_max 2_	44.2	41.7–46.7	44.2	41.9–46.5
Median effective dose_2_	24.6	22.4–26.9	24.6	24.0–25.2
γ_2_	16.3	2.0–30.6	16.7	12.3–22.3
**Suppression Ratio (SR)**				
Median effective dose	26.3	25.4–27.2	26.3	25.7–26.9
γ	18.2	11.5–24.9	18.4	13.3–25.1
